# Beyond training: determinants of nutrition knowledge among athletes in Hungary

**DOI:** 10.3389/fnut.2026.1816064

**Published:** 2026-04-24

**Authors:** Réka Erika Kovács, Szilvia Boros, István Karsai, Gusztáv József Tornóczky

**Affiliations:** 1 Department of Psychology and Health Management, Faculty of Health and Sport Sciences, Széchenyi István University, Gyor, Hungary; 2 National Institute for Sport Medicine, Budapest, Hungary; 3 Physical Education and Exercise Centre, Medical School, University of Pécs, Pécs, Hungary; 4 Institute of Health Promotion and Sport Sciences, Faculty of Education and Psychology, ELTE Eötvös Loránd University, Budapest, Hungary

**Keywords:** ANSKQ-HU, athletes, education, nutrition knowledge, sport nutrition

## Abstract

**Introduction:**

Nutrition knowledge is crucial for athletes' performance and health, yet data on Hungarian athletes are limited.

**Methods:**

This study assessed sport nutrition knowledge and its influencing factors among 1,335 athletes (132 elite, 1,203 recreational; 54.3% male; mean age = 22.74 ± 8.11 years) using the Hungarian Abridged Nutrition for Sport Knowledge Questionnaire (ANSKQ-HU) alongside sociodemographic, sport-related, and perception-based variables. Analyses included Kruskal–Wallis *H*-tests, Mann–Whitney *U*-tests, MANCOVA and Kendall's tau correlations.

**Results:**

Overall, 63.37% of athletes scored in the poor nutrition knowledge category (< 50%). No significant differences were observed by gender, sport level or prior nutrition education. Education level significantly affected sports nutrition knowledge, with higher-educated participants showing better fundamental and overall understanding than those with primary education. These findings were independent of training frequency and age, with medium *post hoc* effect sizes (*d* = 0.31 – 0.35). Weekly training hours and age were weakly correlated with FEP (fundamentals of nutrition, energy requirements of physical activity, and prohibited substances) subscale (*p* < 0.01) and total scores (*p* < 0.01). Participants valuing healthy eating scored higher on FEP (χ^2^ = 30.840, *p* < 0.001, η^2^ = 0.022) and total ANSKQ-HU (χ^2^ = 22.612, *p* < 0.001, η^2^ = 0.015). Access to both nutrition information and dietitian support improved MPE (micronutrients and performance-enhancing sports nutrition) (*U* = 71,395, *Z* = 3.28, *p* = 0.001) and UM (utilization of macronutrients) scores (*U* = 68,231, *Z* = 3.75, *p* < 0.001).

**Discussion:**

Our findings highlight the need for structured nutrition education and dietitian access in sports organizations. Importantly, as the data were collected exclusively from Hungarian athletes, these results reflect country-specific characteristics and should be interpreted within the Hungarian sport and educational context.

## 1 Introduction

The impact of appropriate nutrition on health, recovery, and athletic performance is well established (1–5). Adequate nutrient intake supports training adaptation, optimizes body composition, and reduces the risk of injury and illness. Nevertheless, athletes' dietary behaviors are influenced by a complex combination of social, environmental, psychological, and physiological factors, as well as by their knowledge and perceptions of nutrition (6–8). Individual characteristics such as training experience, competing level, cultural background, and gender may further shape dietary practices and attitudes toward nutrition (9).

Despite the growing availability of qualified professionals in sports nutrition, athletes frequently obtain nutrition-related information from non-professional or unreliable sources, including peers, coaches, and online platforms (10, 11). Previous studies suggest that athletes tend to demonstrate slightly higher levels of nutrition knowledge than non-athletes; however, findings across populations remain inconsistent and often indicate considerable variability in knowledge levels (12, 13). Importantly, higher nutrition knowledge does not always translate into optimal dietary practices, highlighting a persistent gap between knowledge and behavior among athletes (14).

Several factors have been proposed to influence nutrition knowledge in athletic populations. Educational exposure, including formal coursework or structured nutrition education, socioeconomic status has been associated with improved nutrition knowledge in some studies (15–17). Athletes who receive targeted education or professional dietary guidance often demonstrate better understanding of sport-specific nutritional requirements and may adopt healthier dietary behaviors (18–22). However, the relationship between educational attainment and nutrition knowledge is not entirely consistent across studies, suggesting that academic background alone may not fully explain differences in nutritional understanding among athletes (23–25).

In addition to education, athletic experience and training characteristics may also contribute to nutrition knowledge. Greater exposure to structured training environments and competitive sport could increase awareness of nutritional strategies; however, evidence regarding the influence of experience and training volume remains inconclusive (15, 16). Furthermore, access to qualified professionals, such as sports dietitians or nutritionists, appears to play an important role in shaping athletes' knowledge and dietary behaviors. Athletes who receive professional guidance or participate in structured nutrition education programs generally report higher levels of nutrition knowledge and more favorable dietary patterns (10, 26–30). These findings highlight the importance of both educational opportunities and access to credible information sources in promoting adequate nutrition knowledge among athletes.

Although the determinants of nutrition knowledge have been explored in several athletic populations, little is known about the level of nutrition knowledge and its influencing factors among athletes in Hungary. Understanding these determinants may help identify gaps in education and inform targeted interventions aimed at improving nutrition literacy and dietary practices within athletic communities.

Therefore, the aim of this cross-sectional study was to investigate the associations between nutrition knowledge, sociodemographic characteristics, professional guidance, and health-related priorities among Hungarian athletes. We hypothesized that: (1) elite athletes and those with prior nutrition education would demonstrate higher nutrition knowledge scores; (2) athletes with higher educational attainment would achieve higher knowledge scores; and (3) greater athletic experience and weekly training volume would be positively associated with nutrition knowledge. Furthermore, we hypothesized that (4) most respondents would perceive healthy eating, access to reliable nutrition information, and availability of dietetic services as important for optimal sports performance; and (5) access to professional dietary guidance would be associated with higher nutrition knowledge scores.

## 2 Materials and methods

### 2.1 Study design and ethical considerations

Ethical approval for this study was granted by the Research Ethics Committee of the Faculty of Education and Psychology at Eötvös Loránd University on November 13, 2023 (Reference No. 2023/479). Following the translation of the ANSKQ, the Hungarian validation (ANKSQ-HU) and the assessment of nutrition knowledge were conducted using convenience sampling. Participation was voluntary and no financial or other compensation was provided. Elite athletes were recruited at the National Institute for Sport Medicine, while recreational athletes were recruited from sports science students at the Faculty of Education and Psychology, Eötvös Loránd University, and the Faculty of Health and Sports Sciences at Széchenyi István University. Participants were informed about the aims and procedures of the study via an online survey platform. Electronic informed consent was obtained prior to participation by requiring respondents to confirm consent via a checkbox after scanning a QR code and before accessing the questionnaire. Data collection was conducted entirely online using a self-administered format. The same sample was used for both the Hungarian validation of the nutrition knowledge questionnaire (ANKSQ-HU) and the subsequent assessment of nutrition knowledge.

### 2.2 Participants

Participants were recruited from adult athlete population to complete the Hungarian version of the Abridged Nutrition for Sport Knowledge Questionnaire (ANSKQ-HU). Individuals were excluded if they provided incomplete responses, were not athletes, engaged in less than 150 minutes of physical activity per week, or did not specify their sport. Participants were asked to indicate whether they were currently elite athletes or recreational athletes by selecting the appropriate option in a checkbox format. Based on these self-reported responses, the two groups used for the analysis were established.

A total of 1,335 athletes met the inclusion criteria and were included in the analyses. Participants reported involvement in 65 different sports. For the purposes of data analysis, these sports were grouped according to their performance characteristics, as outlined in the validation study of ANSKQ-HU (31); their inclusion here aims to facilitate the interpretation of the results presented below.

### 2.3 Abridged nutrition for sport knowledge questionnaire (ANSKQ) and its Hungarian version (ANSKQ-HU)

The original Nutrition for Sport Knowledge Questionnaire (NSKQ) was developed in 2017 and consisted of 87 items assessing sport nutrition knowledge among athletes (32, 33). To improve feasibility and completion rates, an abridged version, the Abridged Nutrition for Sport Knowledge Questionnaire (ANSKQ), was introduced in 2019 (34).

The ANSKQ contains 35 items covering general and sport-specific nutrition topics, including macronutrients, micronutrients, dietary supplements, weight management, and sports nutrition recommendations. Each correct response is awarded one point, resulting in a maximum score of 35. Scores can be categorized into four levels of nutrition knowledge: poor (< 50%), average (50%−65%), good (66%−75%), and excellent (>75%) (34).

The Hungarian adaptation of the questionnaire (ANSKQ-HU) was developed following established cross-cultural adaptation procedures and in collaboration with the original questionnaire developer. The instrument demonstrated acceptable psychometric properties and a three-factor structure comprising: (1) Fundamentals of nutrition, energy requirements of physical activity, and prohibited substances (FEP); (2) Micronutrients and performance-enhancing sports nutrition (MPE); and (3) Utilization of macronutrients (UM) (31, 35, 36). Detailed information on the validation process and psychometric characteristics of the ANSKQ-HU has been reported previously (31).

In addition to assessing sports nutrition knowledge, the questionnaire collected sociodemographic (e.g., sex, age, place of residence) and sport-related variables (e.g., sport type, weekly training volume, years of experience, and competition level), as well as information on perceived importance of healthy eating, access to nutrition support, and sources of nutrition information. Previous research has also applied the questionnaire in Hungarian athlete populations (37). The questionnaire was administered in accordance with the original administration and scoring procedures described in the ANSKQ validation study (34).

### 2.4 Statistical analysis

Descriptive statistics (means and standard deviations) were calculated to summarize the data. Normality was assessed using skewness and kurtosis indices (38), complemented by the Shapiro–Wilk test. The results indicated that the dependent variables - ANSKQ-HU total score and its three subscales (FEP, MPE, UM) - generally deviated from normal distribution across study groups, whereas the independent variable educational level met the assumption of normality. Accordingly, non-parametric tests were applied in most analyses. For comparisons involving three independent groups, Kruskal–Wallis *H*-tests were conducted. When significant differences were identified, pairwise *post hoc* comparisons were performed using the Mann-Whitney *U*-test with Bonferroni adjustment (α = 0.05/3), resulting in an adjusted significance level of α = 0.017 (39). Associations between continuous variables were examined using Kendall's tau-b correlation coefficients, including relationships among ANSKQ-HU total and subscale scores, weekly training volume, years of sporting experience, and age. In addition, a multivariate analysis of covariance (MANCOVA) was conducted to examine the effect of educational level (three groups) on ANSKQ-HU total and subscale scores, with weekly training volume and age included as covariates due to their observed associations with the total score and the FEP subscale. Bonferroni-adjusted *post hoc* comparisons were performed following significant multivariate effects. Correlation coefficients were interpreted according to Cohen's (1988) criteria: 0.10 ≤ *r* < 0.30 indicating a weak correlation, 0.30 ≤ *r* < 0.50 a moderate correlation, and *r* ≥ 0.50 a strong correlation (40). Effect sizes for the Kruskal–Wallis tests were calculated using eta squared (η^2^), whereas partial eta squared (η^2^) was reported for the MANCOVA (41). Effect size magnitudes were interpreted as η^2^ = 0.01 (small), η^2^ = 0.06 (medium), and η^2^ = 0.14 (large). For pairwise *post hoc* comparisons, Cohen's d was calculated (40, 41), with thresholds defined as *d* < 0.30 (small), 0.30 ≤ *d* < 0.50 (medium), and *d* ≥ 0.50 (large).

All statistical analyses were performed using IBM SPSS Statistics v.30 (IBM Corp., Armonk, NY, USA). Statistical significance was set at α = 0.05, with *p*-values < 0.05 considered statistically significant.

## 3 Results

### 3.1 Sample characteristics

The final sample consisted of 1,335 athletes, including 132 elite athletes and 1,203 recreational athletes. The mean age of the participants was 22.74 years (SD = 8.11), with ages ranging from 18 to 68 years. Regarding gender distribution, 54.3% of respondents (*n* = 725) were male.

Among elite athletes, the highest level of competition most frequently achieved was international for both males (93.3%) and females (92.3%). In contrast, the largest proportion of recreational athletes (41.2%) reported competing at the national level. Additional demographic characteristics, including BMI and weekly training hours, are presented in Supplementary Table 2.

### 3.2 Associations of ANSKQ-HU, gender, nutrition studies and level of competition

**Figure 1:**
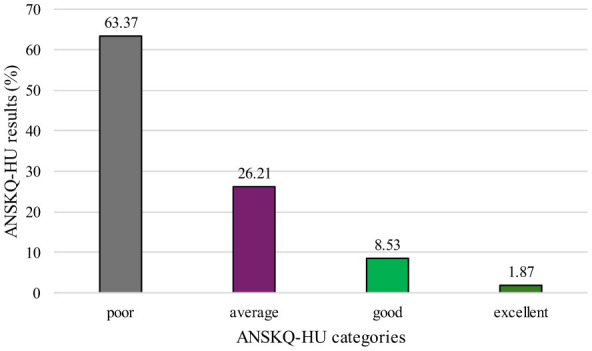
Distribution of ANSKQ-HU results based on knowledge categories ANSKQ-HU: Abriged Nutrition for Sport Knowledge Questionnaire, Hungarian version.

Figure 1 presents the distribution of participants across nutrition knowledge categories (poor, average, good, excellent) as defined by Trakman et al. (32). Nearly two-thirds of participants demonstrated poor nutrition knowledge, while 36.61% achieved at least an acceptable level. Only a small proportion reached the good (8.53%) or excellent (< 2%) categories. Detailed results for the ANSKQ-HU total and subscale scores are reported in the validation study (31). Among all participants, 6.9% (*n* = 93) reported having previously undertaken formal nutrition studies, most frequently through university coursework.

Mann–Whitney *U*-tests revealed no statistically significant differences in nutrition knowledge according to sex, prior nutrition education, or competition level (*p* > 0.05).

### 3.3 Associations of ANSKQ-HU with training characteristics and age

Kendall's tau correlation coefficients were calculated to explore potential relationships between sport nutrition knowledge and individual characteristics, including weekly training hours, years spent in sports, and age (Table 1).

**Table 1:** Kendall's tau correlation coefficients between ANSKQ-HU subscale scores and total score and selected demographic and training variables (*n* = 1335).

Variables	ANSKQ FEP	ANSKQ MPE	ANSKQ UM	ANSKQ-HU
Weekly training hours	0.04^**^	0.01	0.02	0.04^**^
Years in sports	0.03	−0.04	0.007	0.02
Age	0.08^**^	0.02	0.03	0.07^**^

^**^p < 0.01, FEP, Fundamentals of nutrition, energy requirements of physical activity, and prohibited substances; MPE, Micronutrients and performance-enhancing sports nutrition; UM, Utilization of macronutrients; ANSKQ-HU, Abridged Nutrition for Sport Knowledge Questionnaire Hungarian version.

Weak but statistically significant positive associations were observed between weekly training hours and both the FEP subscale and the total ANSKQ-HU score. Similarly, age was positively associated with FEP and the total ANSKQ-HU score. In contrast, years spent in sports were not significantly correlated with any of the questionnaire components (Table 1).

### 3.4 Associations of ANSKQ-HU with educational level

All dependent variables met the assumptions of normality within the educational level groups, and Levene's test confirmed homogeneity of variances (*p* = 0.275–0.879). Therefore, a multivariate analysis of covariance (MANCOVA) was conducted to examine the effect of educational level (primary, secondary, higher education) on sports nutrition knowledge while controlling for age and weekly training volume (Table 2).

**Table 2:** Multivariate analysis of covariance (MANCOVA) examining the effects of age, weekly training hours, and education level on sport nutrition knowledge (*n* = 1335).

Effect	Pillai's trace	F	df (hypothesis, error)	*p*	Partial η^2^
Age	0.003	1.415	3.1325	0.237	0.003
Weekly training hours	0.003	1.178	3.1325	0.317	0.003
Education level	0.014	3.173	6.2652	0.004	0.007

Pillai's Trace is reported as the multivariate test statistic. Age and weekly training hours were included as covariates. Dependent variables were FEP (Fundamentals of nutrition, energy requirements of physical activity, and prohibited substances); MPE, micronutrients and performance-enhancing sports nutrition; UM, utilization of macronutrients; and A-HU total score. Partial η^2^ values represent effect size estimates.

The MANCOVA revealed a significant multivariate effect of educational level on sports nutrition knowledge (Pillai's Trace = 0.014, F(6, 2652) = 3.173, *p* = 0.004), although the associated effect size was small (partial η^2^ = 0.007). Neither age nor weekly training volume showed significant multivariate effects (Table 2).

Follow-up univariate ANCOVAs demonstrated significant differences across educational levels for the FEP subscale, the UM subscale, and the total ANSKQ-HU score (Table 3).

**Table 3:** Results of univariate ANCOVA follow-up tests examining differences in ANSKQ-HU scores by highest educational level (*n* = 1335).

Dependent measure	*p*-value	Partial η^2^	Primary education (*n* = 159, Mean ±SD)	Secondary education (*n* = 956, Mean ±SD)	Higher education (*n* = 220, Mean ±SD)	*Post Hoc* tests summary
FEP	0.026	0.005	6.65 ± 2.51	6.98 ± 2.49	7.54 ± 2.50	HE > PE
MPE	0.497	0.001	0.69 ± 0.99	0.58 ± 0.90	0.57 ± 0.90	Ns
UM	0.005	0.008	2.48 ± 1.39	2.86 ± 1.50	2.75 ± 1.42	SE > PE
A-HU (Total)	0.021	0.006	9.82 ± 3.22	10.42 ± 3.38	10.85 ± 3.48	HE > PE

Education level refers to the highest level of education completed by the participants. Values represent mean ± SD. PE, Primary education; SE, Secondary education; HE, Higher education; FEP, Fundamentals of nutrition, energy requirements of physical activity, and prohibited substances; MPE, Micronutrients and performance-enhancing sports nutrition; UM, Utilization of macronutrients; A-HU, Abridged Nutrition for Sport Knowledge Questionnaire Hungarian version. *Post hoc* comparisons were conducted using Bonferroni correction (*p* < 0.05) based on estimated marginal means. Covariates: age and weekly training hours.

*Post hoc* comparisons indicated that participants with higher education scored significantly higher than those with primary education on the FEP subscale and on the total ANSKQ-HU score. In addition, participants with secondary education scored higher than those with primary education on the UM subscale. No statistically significant differences were observed for the MPE subscale (Table 3).

Bonferroni-adjusted *post hoc* comparisons further confirmed that the significant pairwise differences were primarily observed between the primary education and higher education groups, with higher scores among participants with higher education for both the FEP subscale and the total ANSKQ-HU score (Table 4).

**Table 4:** Bonferroni *post-hoc* tests results between the 3 different educational level groups (*n* = 1335).

Dep. Var.	(I) Edu	(J) Edu	MD (I-J)	SEr	*P*	95% CI		*d*
						**LB**	**UB**	
FEP	PE	SE	−0.309	0.216	0.457	−0.827	0.208	0.13
		HE	−0.778^*^	0.294	0.025	−1.483	−0.074	0.35
	SE	PE	0.309	0.216	0.457	−0.208	0.827	0.13
		HE	−0.469	0.215	0.088	−0.984	0.046	0.22
	HE	PE	0.778^*^	0.294	0.025	0.074	1.483	0.35
		SE	0.469	0.215	0.088	−0.046	0.984	0.22
UM	PE	SE	−0.415^*^	0.128	0.004	−0.721	−0.109	0.26
		HE	−0.409	0.174	0.056	−0.825	0.008	0.19
	SE	PE	0.415^*^	0.128	0.004	0.109	0.721	0.26
		HE	0.006	0.127	1.000	−0.298	0.311	0.08
	HE	PE	0.409	0.174	0.056	−0.008	0.825	0.19
		SE	−0.006	0.127	1.000	−0.311	0.298	0.08
A–HU	PE	SE	−0.630	0.292	0.093	−1.331	0.070	0.18
		HE	−1.099^*^	0.397	0.017	−2.051	−0.146	0.31
	SE	PE	0.630	0.292	0.093	−0.070	1.331	0.18
		HE	−0.468	0.291	0.323	−1.165	0.229	0.13
	HE	PE	1.099^*^	0.397	0.017	0.146	2.051	0.31
		SE	0.468	0.291	0.323	−0.229	1.165	0.13

^*^The mean difference is significant at the 0.05 level. Based on estimated marginal means.

Dep.Var, dependent variable; FEP, Fundamentals of nutrition, energy requirements of physical activity, and prohibited substances; MPE, Micronutrients and performance-enhancing sports nutrition; UM, Utilization of macronutrients; A-HU, Abridged Nutrition for Sport Knowledge Questionnaire Hungarian version; MD, Mean Difference; SEr, Std. Error; CI, Confidence Interval; LB, Lower Bound; UB, Upper Bound; PE, primary education; SE, secondary education; HE, higher education; *d*, Cohen d (effect size estimator).

A significant difference was also identified between the primary and secondary education groups on the UM subscale (Table 4).

Overall, these results suggest an educational gradient in sports nutrition knowledge, although the observed effect sizes remained small.

### 3.5 Association between the ANSKQ-HU and the perception on the importance of healthy eating

Athletes were asked about the importance of healthy eating in their own lives. The majority reported a high perceived importance, with 59% strongly agreeing and 35.5% agreeing. Significant differences in nutrition knowledge were observed across perceived importance of healthy eating (PIHE) groups (Table 5).

**Table 5:** Kruskal-Wallis *H*-test result by perceived importance of healthy eating (PIHE) groups (*n* = 1335).

Variables	No *n* = 72	Agree *n* = 475	Strongly agree *n* = 788	χ^2^	*p*	η^2^
	**Mean** ±**SD**	**Mean** ±**SD**	**Mean** ±**SD**			
FEP	6.77 ± 2.43	6.57 ± 2.49	7.35 ± 2.48	30.840	< 0.001	0.022
MPE	0.81 ± 1.13	0.61 ± 0.88	0.56 ± 0.90	3.731	0.155	0.001
UM	2.44 ± 1.36	2.71 ± 1.56	2.88 ± 1.43	10.009	0.007	0.006
A-HU	9.92 ± 2.87	9.89 ± 3.49	10.79 ± 3.32	22.612	< 0.001	0.015

FEP, Fundamentals of nutrition, energy requirements of physical activity; and prohibited substances; MPE, Micronutrients and performance-enhancing sports nutrition; UM, Utilization of macronutrients; A-HU, Abridged Nutrition for Sport Knowledge Questionnaire Hungarian version; no, not important; agree, important, fully agree, very important.

Athletes who strongly agreed with the importance of healthy eating achieved higher scores in the FEP and UM subscales and in the total ANSKQ-HU score compared with those reporting lower levels of agreement. No significant differences were found for the MPE subscale.

### 3.6 Associations of ANSKQ-HU with the perceived need for nutrition education and dietitian access within sports associations

More than half of participants indicated that sport associations should provide both reliable nutrition information and access to a dietitian, while smaller proportions supported information provision alone or no support.

Significant differences between these groups were observed only for the FEP subscale (Table 6). Athletes who supported the provision of both nutrition information and dietitian access achieved higher FEP scores than those who favored information only or no support.

**Table 6:** Kruskal-Wallis *H*-test result by perceived need for nutrition education and dietitian access groups (*n* = 1335).

Variables	None *n* = 178	d info *n* = 458	d info + diet *n* = 699	χ^2^	*p*	η^2^
	**Mean** ±**SD**	**Mean** ±**SD**	**Mean** ±**SD**			
FEP	6.80 ± 2.62	6.80 ± 2.54	7.24 ± 2.43	9.194	0.010	0.005
MPE	0.66 ± 0.96	0.61 ± 0.91	0.57 ± 0.90	1.497	0.473	0.001
UM	2.78 ± 1.60	2.85 ± 1.47	2.76 ± 1.45	1.131	0.568	0.001
A-HU	10.25 ± 3.82	10.26 ± 3.32	10.57 ± 3.30	3.140	0.208	0.001

FEP, Fundamentals of nutrition, energy requirements of physical activity, and prohibited substances; MPE, Micronutrients and performance-enhancing sports nutrition; UM, Utilization of macronutrients; A-HU, Abridged Nutrition for Sport Knowledge Questionnaire Hungarian version; none, It is not the responsibility of the sports association (no dietitian and no dietetic information); d info, only dietetic information; d info + diet, dietetic information + dietitian.

### 3.7 Association of ANSKQ-HU with the availability of dietetic information and dietitian support

Participants were asked whether they had access to a dietitian and/or reliable sports nutrition information. Only 14.15% reported access to both, whereas more than 65% indicated having access to neither.

Significant differences across diet-support groups were observed for the MPE and UM subscales (Table 7), with higher scores among athletes who reported access to both nutrition information and dietitian support. No significant differences were found for the total ANSKQ-HU score.

**Table 7:** Kruskal-Wallis *H*-test result by dietetical groups (*n* = 1335).

Variables	None *n* = 870	d info *n* = 276	d info + diet *n* = 189	χ^2^	*p*	η^2^
	**Mean** ±**SD**	**Mean** ±**SD**	**Mean** ±**SD**			
FEP	7.21 ± 2.47	6.74 ± 2.52	6.62 ± 2.60	12.511	0.002	0.008
MPE	0.53 ± 0.83	0.66 ± 0.98	0.81 ± 1.13	11.134	0.004	0.007
UM	2.69 ± 1.45	2.89 ± 1.49	3.13 ± 1.51	15.062	< 0.001	0.010
A-HU	10.43 ± 3.40	10.29 ± 3.26	10.56 ± 3.47	0.620	0.733	0.001

FEP, fundamentals of nutrition, energy requirements of physical activity, and prohibited substances; MPE, Micronutrients and performance-enhancing sports nutrition; UM, Utilization of macronutrients; A-HU, ANSKQ-HU; none, no dietitian and no dietetic information; d info, only dietetic information; d info + diet, dietetic information + dietitian.

Additional analyses indicated no significant differences in nutrition knowledge according to sex, prior nutrition education, or competition level, and comparisons across sport disciplines were not feasible due to substantial imbalance in group sizes.

## 4 Discussion

Nearly two-thirds of the athletes (63.37%) demonstrated poor nutrition knowledge according to ANSKQ-HU scores. This finding aligns with international trends reported in previous studies (24, 37). Nevertheless, it highlights the importance of implementing nutrition education interventions as early as possible, targeting not only athletes but also coaches and other key stakeholders (e.g., family members) within the athletes' immediate environment (24, 25).

The first hypothesis was not supported. Although elite athletes scored slightly higher than their recreational counterparts, the difference did not reach statistical significance. Importantly, the mean scores of none of the groups (categorized by gender or level of sport) reached the acceptable minimum threshold of 50%. In contrast, Alahmadi and Albassam reported that Saudi Arabian athletes achieved significantly higher scores than physical activity practitioners (*p* = 0.04) (42). Although the primary focus of this study is athletes, previous research on coaches and support staff was referenced to provide contextual comparison within the wider sporting environment, where these professionals frequently influence athletes' nutrition-related decisions. However, nutrition knowledge in that study was assessed using the Arabic ANSKQ, a culturally adapted and linguistically translated version of the original instrument. The authors emphasized that cultural and dietary specificity—such as high meat consumption and comparatively lower intake of vegetables, dairy products, and fruits in the Saudi Arabian context—may influence both knowledge levels and interpretation of results. In line with previous nutrition studies, Torres-McGehee et al. (43) also reported no statistically significant differences in respondents' nutrition knowledge, assessed using a 19-item questionnaire. This finding is consistent with Andrews et al. (44), who reported no association between nutrition knowledge and prior completion of a nutrition course among mid-major Division I athletes (independent samples *t*-test) (44). However, our results contrast with those of an Irish study, in which athletes who had studied nutrition or dietetics at university level demonstrated significantly higher ANSKQ scores compared to those without such education (independent samples *t*-test, all *p* < 0.05) (10). Comparable findings were reported among NCAA Division III athletes, where regression analysis identified prior nutrition education as a significant predictor of higher ANSKQ scores (45). In our sample, fewer than 10% of participants reported having received formal nutrition education, and only 15 athletes had attended university-level courses. Therefore, the absence of statistically significant differences in our study may partly be explained by the small proportion of participants with formal nutrition training.

The second hypothesis was supported. Education level significantly influenced sports nutrition knowledge (ANSKQ-HU total score and FEP subscale), with participants holding higher educational qualifications demonstrating better fundamental and overall knowledge than those with only primary education (p = 0.021 and *p* = 0.026, respectively). Importantly, this relationship remained significant after controlling for age and training frequency, suggesting that the observed differences are associated with education level rather than age. This finding is consistent with results reported by Smith et al. (10) among Irish athletes. A similar association was observed among coaches in Benghazi, where education level was positively correlated with nutrition knowledge (*r* = 0.2, *p* < 0.05) (46).

Our third hypothesis was partly confirmed, as Kendall's tau correlation coefficient showed statistically significant, weak positive association between weekly training hours, ANSKQ-HU FEP subscale and total scores. Additionally, age was positively associated with FEP and total scores. Bakhtiar et al. similarly reported that, among adolescent trainee athletes (*n* = 260), nutrition knowledge (assessed via a semi-structured, interviewer-administered questionnaire) was significantly associated with age (*p* = 0.007) and training duration (*p* = 0.004) (47). Carey et al. also found that weekly training volume was a significant predictor of food choice (OR [95% CI]: 1.12 [1.06–1.19], *p* < 0.01) (48).

The fourth hypothesis was supported. The majority of participants (59%) strongly agreed, 35.5% agreed, and fewer than 10% disagreed with the statement that healthy eating is important for sports performance. More than half of the sample (52.3%) considered both nutrition education and access to a dietitian to be the responsibility of sports associations; 34.3% expected information provision only, while 14.6% indicated no such expectations. These findings are consistent with previous studies (11, 49–51), which emphasize the need for sports associations and clubs to implement strategies ensuring broad athlete access to qualified sports nutrition professionals.

The fifth hypothesis was also confirmed. A statistically significant association was observed between the availability of nutrition-related information and/or dietitian access and the level of nutrition knowledge. Participants with both access to nutrition information and dietitian support performed better on the MPE and UM subscales, whereas those with nutrition information alone scored higher on the FEP subscale. In all cases, effect sizes were small. Access to sports nutrition professionals, such as dietitians, has been shown to positively influence athletes' nutrition knowledge. Evidence suggests that individualized consultations and structured, evidence-based interventions are associated with improved understanding of dietary principles and more favorable nutrition-related behaviors (52, 53). Although not all studies have directly examined dietitian consultations, the overall body of evidence supports the role of qualified nutrition professionals in enhancing athletes' knowledge and potentially improving performance-related dietary practices.

This study has several strengths that enhance the robustness and relevance of its findings. The large sample size (*n* = 1,335), including both elite and recreational athletes from 65 different sports, provides substantial statistical power across diverse athletic populations. The use of the validated ANSKQ-HU instrument ensures reliable assessment of nutrition knowledge, while the comprehensive collection of sociodemographic, sport-related, and perception-based variables enables multifactorial analysis of potential influencing factors. Furthermore, by examining access to dietitians and nutrition information, the study addresses practical considerations relevant to sports organizations aiming to improve athletes' nutrition knowledge.

Nevertheless, certain limitations should be acknowledged. Data were self-reported, introducing the possibility of reporting bias, and unequal group sizes limited sport-specific analyses. Although some statistically significant differences were identified, effect sizes were generally small, suggesting limited practical significance. Access to a dietitian was assessed dichotomously, without accounting for the frequency, duration, or quality of consultations, which may differentially influence nutrition knowledge. Moreover, the work of dietitians is inherently difficult to quantify, as the transfer of nutrition-related information may occur through multiple channels and its effectiveness depends largely on athletes' receptiveness.

Interestingly, participants without access to nutrition information outperformed others on the FEP subscale. The grouping of items within Factor 1 reflects the structure identified in the exploratory factor analysis and represents a broader construct of foundational sports nutrition knowledge rather than individual question performance. It is possible that some athletes acquired knowledge independently (e.g., through scientific literature or expert media content); however, these sources were not systematically assessed. Although ANSKQ has been widely validated across languages and cultural contexts, differences in dietary guidelines, educational systems, and cultural backgrounds may affect item interpretation. Therefore, comparisons between Hungarian findings and those from other populations should be interpreted cautiously. As participants were recruited from Hungarian institutions, generalizability to athletes in other cultural or organizational settings may be limited. Additionally, comparisons with previous studies were constrained by heterogeneity in measurement tools and study populations. Nevertheless, the present findings contribute valuable insights to the international literature, particularly given the limited data available on Hungarian athletes.

## 5 Conclusion

The present study indicates that a considerable proportion of Hungarian athletes, both elite and recreational, demonstrate limited nutrition knowledge, with only a small percentage achieving good or excellent scores on the ANSKQ-HU. Access to reliable nutrition information and dietitian support was associated with higher scores on specific subscales—particularly micronutrients, performance-enhancing nutrition, and macronutrient utilization—although effect sizes were small. Importantly, even modest effect sizes may be meaningful in the context of nutrition research: when exposures are common and affect large populations, such associations can still have substantial public health implications (49). Additional factors, including education level, weekly training hours, age, and the perceived importance of healthy eating, were also modestly associated with nutrition knowledge. These findings highlight the importance of providing evidence-based nutrition education and ensuring access to qualified nutrition professionals within sports organizations to enhance athletes' nutrition literacy. Further research is warranted to examine the impact of dietitian consultations and structured nutrition interventions on both knowledge acquisition and dietary behaviors across diverse athletic populations.

## Data Availability

The original contributions presented in the study are included in the article/supplementary material, further inquiries can be directed to the corresponding author.
